# Risk of Severe Non AIDS Events Is Increased among Patients Unable to Increase their CD4+ T-Cell Counts >200+/μl Despite Effective HAART

**DOI:** 10.1371/journal.pone.0124741

**Published:** 2015-05-28

**Authors:** Giuseppe Lapadula, Liliane Chatenoud, Andrea Gori, Francesco Castelli, Simona Di Giambenedetto, Massimiliano Fabbiani, Franco Maggiolo, Emanuele Focà, Nicoletta Ladisa, Laura Sighinolfi, Massimo Di Pietro, Angelo Pan, Carlo Torti, Italian MASTER Cohort

**Affiliations:** 1 Clinic of Infectious Diseases, “San Gerardo de’ Tintori” Hospital, Monza, Italy; 2 IRCCS—Istituto di Ricerche Farmacologiche “Mario Negri”, Milan, Italy; 3 University Division of Infectious and Tropical Diseases, University of Brescia, Brescia, Italy; 4 Clinic of Infectious Diseases, “Sacro Cuore” Catholic University of Rome, Rome, Italy; 5 Clinic of Infectious Diseases, Ospedali Riuniti, Bergamo, Italy; 6 Clinic of Infectious Diseases, Ospedale Policlinico, Bari, Italy; 7 Clinic of Infectious Diseases, Ospedale Sant’Anna, Ferrara, Italy; 8 Clinic of Infectious Diseases, Ospedale S.M. Annunziata, Florence, Italy; 9 Clinic of Infectious Diseases, Istituti Ospitalieri, Cremona, Italy; 10 Unit of Infectious Diseases, Department of Medical and Surgical Sciences, University “Magna Graecia”, Catanzaro, Italy; University Hospital Zurich, SWITZERLAND

## Abstract

**Background:**

Immunological non-response (INR) despite virological suppression is associated with AIDS-defining events/death (ADE). Little is known about its association with serious non-AIDS-defining events (nADE).

**Methods:**

Patients highly-active antiretroviral therapy (HAART) with <200 CD4+/μl and achieving HIV-RNA <50 copies/ml within 12 (±3) months were categorized as INR if CD4+ T-cell count at year 1 was <200/μl. Predictors of nADE (malignancies, severe infections, renal failure—ie, estimated glomerular filtration rate <30 ml/min, cardiovascular events and liver decompensation) were assessed using multivariable Cox models. Follow-up was right-censored in case of HAART discontinuation or confirmed HIV-RNA>50.

**Results:**

1221 patients were observed for a median of 3 (IQR: 1.3-6.1) years. Pre-HAART CD4+ were 77/μl (IQR: 28-142) and 56% of patients had experienced an ADE. After 1 year, CD4+ increased to 286 (IQR: 197-387), but 26.1% of patients were INR. Thereafter, 86 nADE (30.2% malignancies, 27.9% infectious, 17.4% renal, 17.4% cardiovascular, 7% hepatic) were observed, accounting for an incidence of 1.83 events (95%CI: 1.73-2.61) per 100 PYFU. After adjusting for measurable confounders, INR had a significantly greater risk of nADE (HR 1.65; 95%CI: 1.06-2.56). Older age (per year, HR 1.03; 95%CI: 1.01-1.05), hepatitis C co-infection (HR 2.09; 95%CI: 1.19-3.7), a history of previous nADE (HR 2.16; 95%CI: 1.06-4.4) and the occurrence of ADE during the follow-up (HR 2.2; 95%CI: 1.15-4.21) were other independent predictors of newly diagnosed nADE.

**Conclusions:**

Patients failing to restore CD4+ to >200 cells/μl run a greater risk of serious nADE, which is intertwined or predicted by AIDS progression. Improved management of this fragile population and innovative therapy able to induce immune-reconstitution are urgently needed. Also, our results strengthen the importance of earlier diagnosis and HAART introduction.

## Introduction

The introduction of highly active anti-retroviral therapy (HAART) among patients formerly naïve to treatment leads to the suppression of HIV replication in most cases.[1] However, a variable proportion of subjects, ranging from 6 to 20%, commonly referred to as “immunological non responders” (INR), fail to achieve a significant immune recovery, as measured by peripheral CD4 cell count, despite virological response to HAART.[2–7]

These patients have been demonstrated to run a greater risk of AIDS progression [6–9] and death, particularly when CD4+ T-cell count is <200/μl.[4,10,11] A recent study has also demonstrated that patients unable to restore their CD4+ T-cell count to >200 cells/μl run a higher risk of death not only due to AIDS-defining diseases, but also due to non AIDS-defining causes.[12]

In a previous study, more non AIDS-defining events (nADE) were observed among patients with a CD4+ T-cell count <200/μl after 2 years of effective HAART than among those with higher CD4+ counts.[5] However, the association was not confirmed in a model adjusted for age, gender and other confounders, probably because patients were included irrespectively of pre-HAART CD4+ T-cell counts, so that those whose CD4+ counts were >200 cells/μl were more likely to obtain a CD4+ above this threshold. Lastly, in a cohort of 3378 patients with various baseline CD4+ T-cell count, lack of immunological recovery (defined as CD4+ count <120% of baseline level) was an independent predictor of clinical progression even in the subgroup of patients with baseline CD4 <200/μl, when a composite measure was considered (AIDS, serious nADE, and all-cause death), but not for serious nADE or death.[13] This study was not focused on patients with advanced stage of infection at baseline, whose risk of AIDS is greater during the first months of HAART[14]. Moreover, percentage increase in CD4+ T-cell count may not be the best marker of immune-reconstitution in patients with low absolute CD4+ T-cell count and its clinical transferability may not be optimal for the sake of clinicians who may prefer CD4+ T-cell count increase above a cut-off. Whether patients who fail to increase their CD4+ T-cell count from below to more than 200 cells/μl are at increased risk of severe nADE is, therefore, still unclear and merits further investigation.

The aim of the present study was to assess whether immune-recovery during virological successful HAART among late-presenters (i.e., patients with pre-HAART CD4+ count <200 cells/μl) is associated with the risk of severe non-AIDS related morbidity and mortality, independently of possible confounders.

## Materials and Methods

### Patient population

Patients were included from the observational Italian MASTER Database Cohort.[15,16] In brief, it is an ongoing prospective multicentre cohort that includes all patients in care for HIV infection in selected major Italian clinical centres. Data are collected using a common electronic database (*HealthNotes* or *NetCare*, Healthware Technology SpA, Salerno, Italy), which is used to manage the everyday activity of the outpatient HIV clinic in each centre. The MASTER cohort is therefore a dynamic database, as new patients presenting to clinical centres are continuously enrolled and patient drop-out rates reflect the real clinical practice. Patients are routinely seen every 3–4 months and demographic, laboratory, clinical and treatment information are collected during each visit. Data from centres are centralized, merged and checked for consistency on a 6-month basis. The database used for the present analysis was frozen in November 2011 and includes data from 8 clinical centres (Bari, Brescia, Bergamo, Cremona, Ferrara, Florence, Monza and Rome).

HIV-1 infected patients who had initiated their first HAART regimen (i.e., including ≥3 antiretroviral drugs) while naïve to antiretroviral therapy between January 1^st^ 1996 and December 31^st^ 2009 were selected from the Italian MASTER Cohort. Other inclusion criteria were: (1) CD4+ T-cell count <200 cells/μl before HAART initiation; (2) Two consecutive HIV plasma viral load <50 copies/ml within the first year (±3 months) of treatment; (3) Age ≥ 18 years. Patients with HIV-2 infection and those with HIV plasma viral load <50 copies/ml before HAART initiation were excluded from the study.

### Outcome measures

The outcome of interest was the occurrence of newly diagnosed serious nADE. Serious nADE included the following: (i) any non AIDS-defining malignancy; (ii) cardiovascular events (acute myocardial infarction, coronary disease requiring invasive procedures, stroke); (iii) severe non-AIDS defining infections (i.e. infections that are potentially life-threatening or require intravenous antibiotic, such as sepsis, episodic pneumonia, endocarditis, meningitis, bacterial arthritis/osteomyelitis, pyelonephritis, cholangitis/cholecystitis, severe skin and soft tissue infections); (iv) hepatic events (hepatocellular carcinoma, decompensated cirrhosis, i.e. variceal bleeding, porto-systemic encephalopathy, refractory ascites, hepatorenal syndrome, portal thrombosis); (v) acute kidney injury (defined as confirmed estimated glomerular filtrate rate [eGFR] <30 ml/min using Modification of Diet in Renal Disease (MDRD) formula [17], or kidney failure requiring dialysis or renal transplantation).

A composite end-point, including nADE, AIDS-defining events [18] and death for any cause, was used as secondary outcome measure.

Serious nADE were considered only if no event of that specific category (e.g., cardiovascular events) had occurred before the study baseline, in a time window covering 1 year before and 1 year after HAART initiation. In addition, episodic pneumonia did not concur to define a newly diagnosed nADE among patients with a previous diagnosis of recurrent bacterial pneumonia. Similarly, an AIDS-defining event was considered only if the patient had not experienced that particular AIDS-related opportunistic infection or neoplasm before.

### Statistical analysis

Uni- and multivariable logistic regression models were conducted to identify variables associated with immunologic non response at 1 year after the initiation of HAART. Predictors of non AIDS-related severe event occurring after year 1 were assessed using Kaplan-Meier estimates and adjusted Cox proportional hazard regression analysis. Follow-up accrued from the first viral load (VL) <50 copies/ml measured after 9–15 months of treatment and was right censored in case of VL >50 copies/ml or missing for ≥180 days, HAART discontinuation, loss to follow-up or (in the analysis of nADE) death. Patients were categorized as INR or immunological responders if their CD4+ T-cell count was <200 or ≥200 cells/μl after 1 year (range 9–15 months) of HAART, respectively.

The following covariates were tested: immunological response at year 1 (INR *versus* immunological responders), age, gender, risk factor for HIV acquisition, previous nADE, previous AIDS-defining event (graded according to its severity in two categories—mild and moderate/severe—as previously described [19]), hepatitis B and C co-infection (defined by serum-reactivity for hepatitis B surface antigen and hepatitis C virus antibody [HCV-Ab], respectively), pre-HAART CD4+ T-cell count, CD4+ count change at year 1 (percentage change), type of HAART and occurrence of AIDS-defining event during the follow-up. Two multivariable models were performed: in the first one, all variables were adjusted for age and gender. In the second model, estimates were adjusted for all the variables significantly associated with the outcome in the first model plus age, gender and CD4+ T-cell count percentage change (considered to be clinically significant). When more than one event had occurred in a single patient, the time to the event was defined as the time between baseline and the first occurrence of any of the events considered.

The same models and covariates (with the exception of the occurrence of AIDS-defining events during the follow-up) were used in the secondary analysis exploring predictors of the composite outcome (serious nADE, AIDS-defining event or death).

All statistical analyses were performed using SAS 9.2 statistical software (SAS Institute Inc., Cary, NC, USA, 2008). All P-value presented are two sided and a P-value <0.05 indicated conventional statistical significance.

### Ethics Statement

Patients included in the MASTER cohort study provide, at enrolment, written informed consent to include their clinical and biological data in the MASTER database for scientific purposes. The data are anonymized and the database is hosted in the “Fondazione MISI’s” headquarter, in compliance with current Italian regulations. The study was approved by the Ethical Committee of the Hospital “Spedali Civili”, Brescia (Coordinating Centre) and those of the following Institutions: University Hospital of Ferrara, Ferrara; AO Papa Giovanni XXIII, Bergamo; Ospedale Policlinico—University of Bari, Bari; San Gerardo de’ Tintori" Hospital, Monza; Hospital of Cremona, Cremona; ‘‘Santa Maria Annunziata” Hospital, Firenze; “Sacro Cuore” Catholic University, Roma.

## Results

### Patient characteristics

Among 1869 patients initiating HAART with a CD4+ T-cell count <200 cells/μl in the MASTER Cohort, 164 patients were not included because followed for <12 months, 477 because did not have two consecutive HIV plasma viral load <50 copies/ml within the first year of treatment and 7 because lacked of CD4+ T-cell count determination at month 12. The remaining 1221 patients were included and studied over a median of 3 years (Inter-quartile range [IQR] 1.3–6.1), accounting for a total of 4,708 person-years of follow-up (PYFU). The median age at treatment initiation was 41.2 years; the youngest patient was 18 and the oldest 75 years old. Median CD4+ T-cell count at the time of HAART initiation was 77 (IQR 28–142) cells/μl and 56% of the patients had already experienced one or more AIDS-defining illnesses before study baseline.

After 1 year of treatment, median CD4+ counts had increased to 286 (IQR 197–387) cells/μl, but 319 patients (26%) had a CD4+ count below 200 cells/μl and were, therefore, classified as INR.

Characteristics of the patients, overall and stratified by immunological response after 1 year of treatment, are shown in Table 1.

**Table 1:** Patient characteristics.

Characteristic	INR(N = 319)	IR(N = 902)	Total(N = 1221)
Age, years [Median(IQR)]	42.1 (36.8–49.7)	38.9 (33.6–45.7)	41.2 (34.5–46.6)
Male gender [N(%)]	256 (80.3)	665 (73.7)	921 (75.4)
Italian born [N(%)]	274 (85.9)	744 (82.5)	1018 (83.4)
Route of HIV transmission [N(%)]			
Intravenous drug use	88 (27.6)	139 (15.4)	227 (18.6)
Homosexual intercourse	38 (11.9)	166 (18.4)	204 (16.7)
Heterosexual intercourse	151 (47.3)	470 (52.1)	621 (50.9)
Other/Unknown	42 (13.2)	127 (14.1)	169 (13.8)
AIDS diagnosis before study baseline [N(%)]			
Mild AIDS	109 (34.2)	272 (30.2)	381 (31.2)
Moderate/severe AIDS	103 (32.3)	203 (22.5)	306 (25.1)
Serious non AIDS-defining events before study baseline	26 (8.2)	76 (8.4)	102 (8.4)
Calendar year at HAART initiation [N(%)]			
1997–2000	77 (24.2)	174 (19.3)	251 (20.7)
2001–2005	130 (40.8)	365 (40.5)	495 (40.6)
2006–2010	112 (35.0)	363 (40.2)	475 (38.9)
HCV-Ab [N(%)]			
Negative	134 (42.0)	542 (60.1)	676 (55.4)
Positive	77 (24.1)	139 (15.4)	216 (17.7)
Unknown	108 (33.9)	221 (24.5)	329 (26.9)
HBsAg [N(%)]			
Negative	180 (56.4)	563 (62.4)	743 (60.9)
Positive	15 (4.7)	32 (3.5)	47 (3.8)
Unknown	124 (38.9)	307 (34.0)	431 (35.3)
Type of HAART			
NNRTI-based	92 (28.8)	297 (32.9)	389 (31.9)
Single PI-based	93 (29.1)	203 (22.5)	296 (24.2)
Boosted PI-based	130 (40.8)	384 (42.6)	514 (42.1)
Other	4 (1.3)	18 (2.0)	22 (1.8)
Pre-HAART CD4+, cells/μl [Median(IQR)]	34 (15–76)	101 (43–158)	77 (28–142)
CD4+ at year 1 (study baseline), cells/μl [Median(IQR)]	148 (116–176)	342 (268–438)	286 (197–387)
CD4+ change at year 1, cells/μl [Median(IQR)]	96 (61–129)	242 (176–341)	195 (121–302)
Duration of follow-up, years [Median(IQR)]	3 (1.4–6.7)	3 (1.3–5.8)	3 (1.3–6.1)
AIDS-defining event during follow-up [N(%)]	27 (8.4)	44 (4.9)	71 (5.8)
Severe non AIDS-defining event during follow-up [N(%)]	37 (11.6)	49 (5.4)	86 (7.0)

List of abbreviations: AIDS, acquired immunodeficiency syndrome; HAART, highly-active antiretroviral treatment; HBsAg, hepatitis B surface antigen; HCV-Ab, hepatitis C virus antibodies; HIV, human immunodeficiency virus; INR, immunological non-responders; IQR, interquartile range; IR, Immunological responders; NNRTI, non-nucleoside reverse transcriptase inhibitor; PI, protease inhibitor.

During the subsequent follow-up, 272 (85.3%) INR had at least one determination of CD4+ T-cell count ≥200 cells/μl, whilst 47 (14.7%) remained below this threshold throughout the follow-up.

### Predictors of immunological non-response

Applying a logistic regression model adjusted for age and gender, the following factors were associated with a higher risk of INR after 1 year of treatment: older age (per year increase, OR 1.03, 95%CI 1.02–1.05), intravenous drug use (IVDU) as HIV route of infection (*versus* heterosexual route, OR 1.92, 95%CI 1.37–2.70), hepatitis C co-infection (OR 2.26, 95%CI 1.6–3.19), previous AIDS event (mild event: OR 1.75, 95%CI 1.25–2.43; moderate severe event: OR 2.15, 95%CI 1.56–3.03) and use of single PI-based HAART (*versus* NNRTI-based HAART, OR 1.53, 95%CI 1.09–2.16). Conversely, initiating HAART in latest years (*versus* 1996–2000, 2001–2005 OR 0.749, 95%CI 0.53–1.05; 2006–2010 OR 0.62, 95%CI 0.44–0.88; Chi-squared for trend <0.05) and higher pre-HAART CD4+ T-cell counts (per 10 cells/μl increase, OR 0.85, 95%CI 0.83–0.87) were associated with lower risk of INR.

In multivariable logistic regression models, older age (per year increase, adjusted OR [aOR] 1.04, 95%CI 1.02–1.06), IVDU route of infection (*versus* heterosexual route, aOR 2.45, 95%CI 1.68–3.58) and pre-HAART CD4+ T-cell count (per 10 cells/μl increase, aOR 0.84, 95%CI 0.82–0.87) were significantly associated with INR after 1 year of treatment, after adjustment for each other and for gender, history of previous AIDS-defining events, calendar year at treatment initiation and type of HAART initiated. The association between hepatitis C seroreactivity and a higher risk of INR was confirmed in a separate multivariable logistic regression model not including risk factor for HIV acquisition (aOR 2.74, 95%CI 1.87–4.01).

### Events description

Overall 1620 clinical events were observed since 1 year before HAART initiation: 983 before HAART (901 AIDS-defining and 86 serious nADE), 399 during the first year of treatment (317 AIDS-defining and 82 serious nADE) and 238 during the subsequent follow-up (112 AIDS-defining, 111 serious nADE and 15 deaths). Full description of all observed events is included in S1 Table.

For the sake of the present analysis, only the first event occurring after year 1 was retained. Therefore, 86 serious nADE were considered, accounting for an incidence of 1.83 (95%CI 1.73–2.61) events per 100 PYFU. Of them, 26 (30.2%) were non AIDS-defining malignancies, 15 (17.4%) cardiovascular events, 24 (27.9%) severe infections, 15 (17.4%) renal and 6 (7%) hepatic events.

During the same follow-up, excluding the events that occurred after a serious nADE, 71 AIDS-defining events and 6 deaths were observed. A detailed description of the events observed during the study time-window and their frequency among immunological responders and INR is shown in Table 2.

**Table 2:** Severe non AIDS-related events observed during the follow-up (overall results and stratified by immunological response at year 1).

Event	Total (N = 1221)	INR (N = 319)	IR (N = 902)
Malignancies	26 (2.1%)	8 (2.5%)	18 (2%)
Lung carcinoma	2	1	1
Cutaneous carcinoma	4	1	3
Melanoma	2	1	1
Anal carcinoma	2	1	1
Hodgkin Lymphoma	2	0	2
Other	14	4	10
Severe infections	24 (2%)	9 (2.8%)	15 (1.7%)
Episodic Pneumonia	17	7	10
Encephalitis/Meningitis	2	1	1
Sepsis	2	0	2
Other severe infections	3	1	2
Cardiovascular events	15 (1.2%)	5 (1.6%)	10 (1.1%)
Acute myocardial infarction	12	3	9
Cerebrovascular accident	3	2	1
Renal events	15 (1.2%)	10 (3.1%)	5 (0.5%)
Confirmed eGFR <30 ml/min	14	9	5
Renal failure requiring dyalisis	1	1	0
Hepatic events	6 (0.5%)	5 (1.6%)	1 (0.1%)
Decompensated cirrhosis	4	3	1
Hepatocellular carcinoma	2	2	0
AIDS-defining events	71 (5.8%)	27 (8.5%)	44 (4.9%)
Recurrent bacterial pneumonia	12	6	6
Esophageal candidiasis	11	2	9
Non-Hodgkin lymphoma	7	3	4
Tuberculosis	5	1	4
Cytomegalovirus disease	5	2	3
Other AIDS-defining illnesses	31	13	18
Deaths	9 (0.7%)	5 (1.6%)	4 (0.4%)
Violent cause	2	1	1
Lactic acidosis and acute renal failure	1	1	0
Decompensated diabetes and acute renal failure	1	0	1
Intestinal obstruction	1	0	1
Cancer (diagnosed before study entry)	2	1	1
Unspecified AIDS-cause	2	2	0
**Total**	**166 (13.6%)**	**69 (21.6%)**	**97 (10.7%)**
**Serious non AIDS-defining events**	**86 (7%)**	**37 (11.6%)**	**49 (5.4%)**
**AIDS-defining events**	**71 (5.8%)**	**27 (8.5%)**	**44 (4.9%)**
**Deaths**	**9 (0.7%)**	**5 (1.6%)**	**4 (0.4%)**

List of abbreviations: AIDS, acquired immunodeficiency syndrome; eGFR, estimated glomerular filtration rate; INR, immunological non-responders; IR, Immunological responders.

### Predictors of serious non-AIDS related events

**Fig 1:**
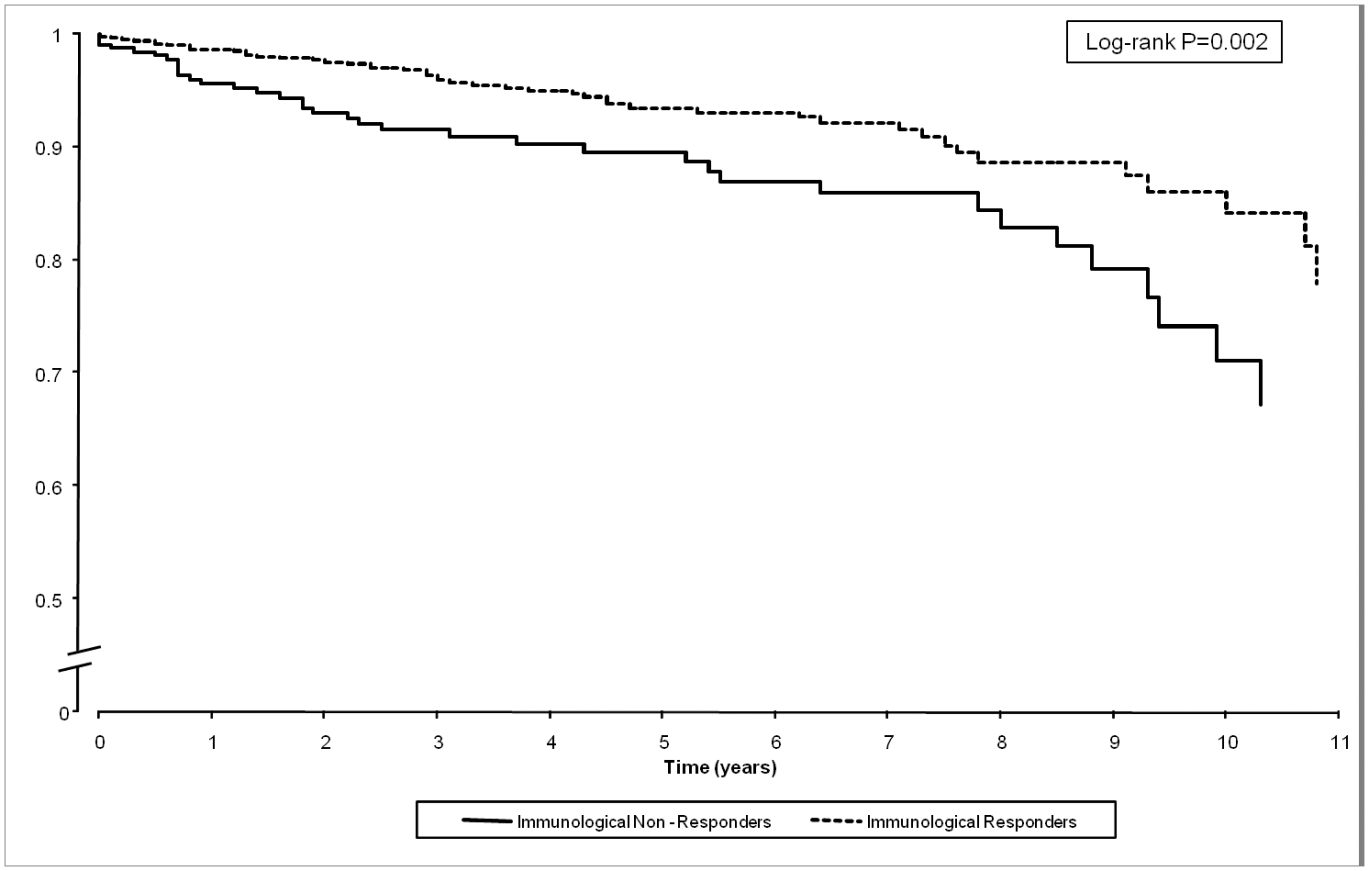
Proportion of patients remaining free from severe non AIDS-related event. List of abbreviations: AIDS, acquired immunodeficiency syndrome; HAART, Highly-active antiretroviral therapy.

Fig 1 shows Kaplan-Meier survival plot of risk of developing serious nADE amongst INR and immunological responders at year 1.

Applying a Cox regression model adjusted for age and gender, INR were at significantly higher risk of nADE than those with >200 CD4+ T cells/μl after 1 year of treatment (HR 1.82; 95%CI 1.18–2.82). Other factors significantly associated with nADE were older age (per year, HR 1.03; 95%CI 1.01–1.05), IVDU as risk factor for HIV acquisition (HR 1.72; 95%CI 1.05–2.82), hepatitis C co-infection (HR 2.32; 95%CI 1.32–4.06), having had a previous nADE (HR 2.55; 95%CI 1.26–5.18) or a previous AIDS-defining event (for mild events: HR 1.82; 95%CI 1.03–3.22; for moderate/severe events: HR 1.34; 95%CI 0.73–2.46) and the occurrence of AIDS-defining event during the follow-up (HR 2.64; 95%CI 1.39–5.01). Due to high correlation (63%) between IVDU and hepatitis C serostatus, only IVDU was included in a second multivariate Cox model, where adjustments for all factors significantly associated with the outcome in the previous model and those considered to be clinically significant (i.e., age, gender and CD4+ percentage change after 1 year of HAART) were included. In this analysis, the following factors were significantly and independently associated with a higher risk of nADE: immunological response at year 1 (INR *vs*. full responders, HR 1.61, 95%CI 1.03–2.51), older age (per year, HR 1.03, 95%CI 1.01–1.05), previous serious nADE (HR 2.52, 95%CI 1.22–5.22) and occurrence of AIDS defining event during the follow-up (HR 2.07; 95%CI 1.08–3.99). The association between hepatitis C serostatus and increasing risk of severe nADE was confirmed in a separate multivariable model, not including risk factor for HIV acquisition (HR 1.98; 95% 1.11–3.53). When CD4+ percentage change after 1 year of HAART was considered in a model including immunological response at year 1, higher CD4+ increases were only marginally associated with lower risk of nADE (per percent point increase, HR 0.98; 95%CI 0.96–1.00). Results of Cox proportional hazard models are shown in Table 3.

**Table 3:** Multivariable Cox Proportional Hazard Models for Time to severe non-AIDS related event.

Variable	**First Model** ^**1**^ **Hazard Ratio (95%CI)**	**Second Model** ^**2**^ **Hazard Ratio (95%CI)**
Immunological non response	1.82 (1.18–2.82)	1.61 (1.03–2.51)
Age (per year)	1.03 (1.01–1.05)	1.03 (1.01–1.05)
Male gender	1.09 (0.67–1.79)	1.32 (0.79–2.20)
Italian born	1.12 (0.54–2.31)	1.41 (0.67–3.00)
Route of HIV transmission—Intravenous drug use	1.72 (1.05–2.82)	1.45 (0.87–2.42)
Previous AIDS-defining event		
Mild events	1.82 (1.03–3.22)	1.77 (0.99–3.15)
Moderate/severe events	1.34 (0.73–2.46)	1.34 (0.72–2.53)
Previous severe non AIDS-defining event	2.55 (1.26–5.18)	2.52 (1.22–5.22)
HBsAg positivity	1.21 (0.38–3.92)	0.98 (0.30–3.20)
HCV-Ab positivity	2.32 (1.32–4.06)	1.98 (1.11–3.53)*
Type of HAART		
NNRTI-based	1.00	1.00
Single PI-based	1.61 (0.95–2.72)	1.44 (0.84–2.47)
Boosted PI-based	0.77 (0.42–1.4)	0.79 (0.43–1.44)
Other	1.78 (0.42–7.61)	1.83 (0.42–7.96)
Pre-HAART CD4+ (per 10 cells/μl)	1.02 (0.99–1.06)	1.07 (1.02–1.12)
CD4+ percentage change at year 1(per percent point)	0.98 (0.96–1.00)	0.98 (0.96–1.00)
Occurrence of AIDS-defining event during follow-up	2.64 (1.39–5.01)	2.07 (1.08–3.99)

List of abbreviations: AIDS, acquired immunodeficiency syndrome; CI, confidence interval; HAART, highly-active antiretroviral treatment; HBsAg, hepatitis B surface antigen; HCV-Ab, hepatitis C virus antibodies; HIV, human immunodeficiency virus; INR, immunological non-responders; IR, Immunological responders; NNRTI, non-nucleoside reverse transcriptase inhibitor; PI, protease inhibitor.

^1^ The first model was adjusted for age and gender.

^2^ The second model was adjusted for age, gender, intravenous drug use as route of HIV transmission, pre-HAART CD4+ T-cell count, occurrence of AIDS-defining events and immunological response (Immunological non responders at year 1 *versus* responders). Occurrence of AIDS-defining event during follow-up was considered as time-dependent variable.

* Estimates for HCV-Ab positivity were not adjusted for intravenous drug use as route of HIV transmission, due to high correlation.

An independent association between INR and risk of nADE events was confirmed after stratification for pre-HAART CD4+ T-cell count (adjusted HR 2.39, 95%CI 1.44–3.86 and HR 1.84, 95%CI 1.03–3.29 for patients with < and ≥77 cells/mm^3^ before HAART, the median baseline CD4+ count).

In order to explore whether the increased risk of nADE among INR could have been influenced by a higher rate of AIDS events in this subgroup of patients, all models were re-run after excluding those patients in whom an AIDS-defining event had occurred before a serious nADE. In these analyses, INR remained significantly associated with higher risk of severe nADE either in univariable or in multivariable Cox regression analysis (HR 1.78, 95%CI 1.12–2.84 and HR 1.73, 95%CI 1.08–2.79, respectively).

### Predictors of death, AIDS-defining and serious non AIDS-defining events

Similar results were obtained when the composite end-point of death, AIDS-defining and serious nADE was used as outcome measure. Using multivariable Cox regression analysis, the following variables were associated with increased risk of clinical progression: INR at year 1, having experienced a previous AIDS-defining event, HCV-Ab positivity. Conversely, higher CD4+ change at year 1, compared with baseline, resulted to be borderline protective. Results of this analysis are shown in Table 4.

**Table 4:** Multivariable Cox Proportional Hazard Models for Time to first AIDS-defining event or severe non AIDS-defining event or death.

Variable	First Model^1^ Hazard Ratio (95%CI)	Second Model^2^ Hazard Ratio (95%CI)
Immunological non response	1.88 (1.34–2.63)	1.67 (1.18–2.35)
Age (per year)	1.01 (0.99–1.03)	1.01 (0.99–1.02)
Male gender	1.09 (0.74–1.59)	1.27 (0.87–1.87)
Italian born	1.06 (0.63–1.78)	1.36 (0.79–2.32)
Route of HIV transmission—Intravenous drug use	1.52 (1.04–2.22)	1.21 (0.82–1.80)
Previous AIDS-defining event		
Mild events	1.94 (1.25–3.01)	1.93 (1.24–3.01)
Moderate/severe events	1.54 (0.96–2.46)	1.46 (0.90–2.37)
Previous severe non AIDS-defining event	2.07 (1.7–5.18)	2.86 (1.62–5.04)
HBsAg positivity	1.73 (0.84–3.58)	1.44 (0.69–3.01)
HCV-Ab positivity	1.56 (1.00–2.44)	1.20 (0.76–1.90)*
Type of HAART		
NNRTI-based	1.00	1.00
Single PI-based	2.11 (1.41–3.15)	1.96 (1.30–2.97)
Boosted PI-based	0.82 (0.52–1.29)	0.84 (0.53–1.32)
Other	1.95 (0.69–5.48)	2.11 (0.74–6.03)
Pre-HAART CD4+ (per 10 cells/μl)	0.99 (0.97–1.03)	1.03 (0.99–1.07)
CD4+ percentage change at year 1(per percent point)	0.98 (0.97–0.99)	0.98 (0.97–1.00)

List of abbreviations: AIDS, acquired immunodeficiency syndrome; CI, confidence interval; HAART, highly-active antiretroviral treatment; HBsAg, hepatitis B surface antigen; HCV-Ab, hepatitis C virus antibodies; HIV, human immunodeficiency virus; INR, immunological non-responders; IR, Immunological responders; NNRTI, non-nucleoside reverse transcriptase inhibitor; PI, protease inhibitor.

^1^ The first model was adjusted for age and gender.

^2^ The second model was adjusted for age, gender, intravenous drug use as route of HIV transmission, pre-HAART CD4+ T-cell count, occurrence of AIDS-defining events and immunological response (Immunological non responders at year 1 *versus* responders). Occurrence of AIDS-defining event during follow-up was considered as time-dependent variable

* Estimates for HCV-Ab positivity were not adjusted for intravenous drug use as route of HIV transmission, due to high correlation.

## Discussion

Notwithstanding efforts to promote early HIV testing, 20 to 40% of patients entering in care for HIV in Western Countries present with advanced HIV disease, i.e. with a CD4+ count below 200 cells/μL.[20–24] Failure to restore CD4+ count above this threshold, despite virological effective HAART, is a relatively common finding, which has been associated with increased risk of AIDS progression and death.[4,5,9,12] Whether a lack of short-term CD4+ increase, despite suppression of HIV replication, is also associated with a greater risk of non AIDS-related morbidities is still unclear.

In our cohort, failure to increase CD4+ T-cell count from below to more than 200 cells/μl after 1 year of effective HAART increased the subsequent risk of severe non AIDS-related event by 65%. These results are in agreement with a recent study that demonstrated that patients unable to restore their CD4+ count to >200 cells/μl after 3 years of viral suppression run a higher risk of death due to non AIDS-defining causes (particularly non AIDS-defining cancer and liver-related). This study, however, could not explore the possible association between INR and non-fatal events.[12] A previous study had suggested an association between nADE and CD4+ T-cell count <200/μl measured after 2 years of HAART, but could not demonstrate that this association was independent from age, gender and other possible confounders. Moreover, the study included only a minority of patients whose pre-HAART CD4+ count was <200 cells/μl.[5] The large number of patients enrolled in our study and the long follow-up provided the opportunity to specifically evaluate the risk of nADE in patients who had initiated HAART at CD4+ T-cell count <200/μl and to demonstrate that it was associated with incomplete CD4+ recovery, despite virological suppression. Moreover, thank to the relatively high number of events observed, it was possible to run a reliable multivariable model and to demonstrate that the association was independent from measurable confounders. Our findings underlines the need for innovative treatment strategies aimed at improving CD4+ recovery among patients presenting to care with very low CD4+ counts and with sub-optimal immune-recovery after HAART.

When change of CD4+ T-cell count at year 1 compared to pre-HAART levels, instead of the absolute CD4+ count at the same time-point, was used as a covariate, only a marginal, non-statistically significant association with serious nADE was found. Similarly, pre-HAART CD4+ counts were not associated with risk of serious nADE to a statistically significant extent. Previous studies suggested that absolute CD4+ cell count measurement at certain time points after HAART initiation is a more solid prognostic indicator of AIDS clinical progression than CD4+ change from baseline.[25] Our results suggest that this could also be the case for prediction of severe, non AIDS-defining morbidities. Taken together, these findings indicate that clinically important immunological response is likely to be better defined in terms of absolute post-HAART CD4+ cell counts, rather than change from baseline in patients with an advanced immune-depletion.

Interestingly enough, patients developing a new AIDS defining event were shown to have a 2-fold increased risk to be diagnosed with a severe nADE subsequently. Therefore, the increased risk of nADE observed in INR patients could be partially (but not completely) due to their greater risk of developing new AIDS events. It has to be ascertained whether a condition of pre-existing immune dysfunction, immune activation or persistent inflammation [26–29] predispose to both events or the occurrence of AIDS is in the causal pathway leading to nADE. In either case, our results suggest the importance of monitoring patients with AIDS on HAART for the risk of subsequent nADE events, persisting for more than 5 years notwithstanding suppression of HIV RNA induced by HAART. At the same time, although in our patients morbidity and mortality due to severe non AIDS-related events exceeded that due to AIDS-related causes (confirming and further extending previous observations among HIV-infected subjects with relatively preserved CD4+ cell counts [30–32]), prevention of AIDS and earlier HAART remain a priority.

In our cohort, positive HCV-Ab testing was associated with a greater risk of INR. Whether this effect is directly attributable to hepatitis C virus co-infection or to other associated conditions is still debated. A previous study suggested that HCV-Ab positive patients are more likely to have an incomplete CD4+ restoration [33], whereas others suggested that previous intravenous drug use, but not HCV co-infection, is associated with suboptimal CD4 response to HAART. [3,11] Similarly, results on the effect of ongoing hepatitis C virus replication on CD4+ T-cell recovery are conflicting. [34,35] Either way, our study showed that patients with positive HCV-Ab had an almost doubled risk of serious nADE. This finding is not surprising, because hepatitis C virus infection is associated not only with an increased risk of liver-related events, but also with a greater risk of cardiovascular and renal events [36,37], and support the indication for early treatment of HCV infection among patient with low CD4 T-cell counts, which is now more feasible thanks to the availability of highly-effective, all-oral, short-course regimens.[38]

Our study has some limitations that merit to be acknowledged. First, the definition of nADE included a number of different comorbid diseases, which are heterogeneous in terms of pathogenesis. The use of this composite end-point, nonetheless, is widely accepted in the literature.[5,13,39] Unfortunately, our study was not designed nor powered enough to study single items of the composite endpoint, so we can not exclude that the association we found could have been driven by some of its components. Second, we defined INR basing on CD4+ T-cell count after 1 year of effective HAART. This definition may differ from previously adopted definitions and 1 year could be an insufficient time to observe complete CD4+ count restoration. Bearing in mind that no consensus definition exist for INR, our choice was based on some considerations: short-term response to HAART has been previously demonstrated to be a solid prognostic indicator for HIV-infected patients initiating HAART and the threshold of 200 CD4+/mm^3^ is widely accepted to define HIV-related immunedepression.[40] In addition, the evaluation of a CD4+ count at a definite time-point, rather than the comparison of current CD4+ T-cell count with pre-HAART levels, is a simple way to assess immune response in those initiating HAART and can be easily transferred to clinical practice. Third, not all contributing centres linked their data with AIDS and cancer registries and with local hospital records. In addition, information on nADE before inclusion in the MASTER cohort could be incomplete. Therefore, we can not exclude that, in some cases, outcomes could have been underestimated. Conversely, due to the non-spontaneous nature of follow-up visits, it is unlikely that event reporting was more frequent among patients with more compromised conditions, such as AIDS-presenters or INR (detection bias). Eventually, we were not able to adjust for some unmeasured confounders (e.g., smoking or other comorbidities, such as diabetes or dyslipidemia) that may predispose to severe nADE.

In conclusion, our results indicate that increasing CD4+ T-cells >200/μl decreases the risk of severe nADE. Even among those with severe immune-suppression, non-AIDS events risk is greater than risk of AIDS, once patients are started on effective HAART. Interestingly, occurrence of AIDS is intertwined (and possibly predict) the risk of severe nADE events.

## Supporting Information

S1 Table Description of the AIDS-defining events and of the serious non AIDS-defining events observed. (DOCX)
