# Supplementary material for: Risk of Severe Non AIDS Events Is Increased among Patients Unable to Increase their CD4+ T-Cell Counts >200+/μl Despite Effective HAART
Source: PLoS One. 2015 May 28;10(5):e0124741. doi: 10.1371/journal.pone.0124741 (PMC4447446; doi:10.1371/journal.pone.0124741)
Supplement: S1 Table — (DOCX) [file pone.0124741.s001.docx]

**S1 Table**

| **Type of Event** | **Time window** | | | | | | **Total** | |
| --- | --- | --- | --- | --- | --- | --- | --- | --- |
|  | Within 1 year before HAART | | During the 1st year of HAART | | After year 1 (study time window) | |  |  |
|  | **N** | **%** | **N** | **%** | **N** | **%** | **N** | **%** |
| Severe infection | 50 | 5.1% | 14 | 3.5% | 27 | 11.3% | 91 | 5.6% |
| Non AIDS-defining neoplasm | 15 | 1.5% | 7 | 1.8% | 30 | 12.6% | 52 | 3.2% |
| Cardiovascular disease | 11 | 1.1% | 3 | 0.8% | 19 | 8.0% | 33 | 2.0% |
| Liver-related events | . | . | . | . | 14 | 5.9% | 14 | 0.9% |
| Renal events | 6 | 0.6% | 9 | 2.3% | 21 | 8.8% | 36 | 2.2% |
| Death | . | . | . | . | 15 | 6.3% | 15 | 0.9% |
| AIDS HIV-related Encephalopathy | 22 | 2.2% | 16 | 4.0% | 7 | 2.9% | 45 | 2.8% |
| AIDS Recurrent Salmonella Septicemia | 3 | 0.3% | . | . | . | . | 3 | 0.2% |
| AIDS Invasive Cervical Cancer | 3 | 0.3% | 3 | 0.8% | 1 | 0.4% | 7 | 0.4% |
| AIDS Esophageal Candidiasis | 104 | 10.6% | 50 | 12.5% | 20 | 8.4% | 174 | 10.7% |
| AIDS Extrapolmonary Criptococcosis | 16 | 1.6% | 5 | 1.3% | 2 | 0.8% | 23 | 1.4% |
| AIDS Chronic Intestinal Cryptosporidiosis | 6 | 0.6% | 4 | 1.0% | 1 | 0.4% | 11 | 0.7% |
| AIDS Herpes simplex chronic ulcers | 1 | 0.1% | 3 | 0.8% | . | . | 4 | 0.2% |
| AIDS Chronic Intestinal Isosporiasis | 1 | 0.1% | 3 | 0.8% | . | . | 4 | 0.2% |
| AIDS Extrapulmonary Histoplasmosis | . | . | 1 | 0.3% | . | . | 1 | 0.1% |
| AIDS Non-Hodgkin Lymphoma | 13 | 1.3% | 7 | 1.8% | 11 | 4.6% | 31 | 1.9% |
| AIDS Cytomegalovirus Disease | 442 | 45.0% | 71 | 17.8% | 6 | 2.5% | 519 | 32.0% |
| AIDS Nontubercular Mycobacteriosis | 14 | 1.4% | 41 | 10.3% | 4 | 1.7% | 59 | 3.6% |
| AIDS Microsporidiosis | 2 | 0.2% | 1 | 0.3% | . | . | 3 | 0.2% |
| AIDS Progressive Multifocal Leukoencephalopathy | 2 | 0.2% | 5 | 1.3% | . | . | 7 | 0.4% |
| AIDS Recurrent bacterial pneumonia | 138 | 14.0% | 57 | 14.3% | 16 | 6.7% | 211 | 13.0% |
| AIDS Kaposi Sarcoma | 24 | 2.4% | 28 | 7.0% | 3 | 1.3% | 55 | 3.4% |
| AIDS Toxoplasmosis of the Brain | 29 | 3.0% | 21 | 5.3% | 4 | 1.7% | 54 | 3.3% |
| AIDS Tuberculosis | 40 | 4.1% | 24 | 6.0% | 7 | 2.9% | 71 | 4.4% |
| AIDS Wasting Syndrome | 22 | 2.2% | 6 | 1.5% | 5 | 2.1% | 33 | 2.0% |
| AIDS Other | 19 | 1.9% | 20 | 5.0% | 25 | 10.5% | 64 | 4.0% |
| **Total** | **983** | **60.7%** | **399** | **24.6%** | **238** | **14.7%** | **1620** | **100.0%** |
